# Characterizing informative sequence descriptors and predicting binding affinities of heterodimeric protein complexes

**DOI:** 10.1186/1471-2105-16-S18-S14

**Published:** 2015-12-09

**Authors:** Yerukala Sathipati Srinivasulu, Jyun-Rong Wang, Kai-Ti Hsu, Ming-Ju Tsai, Phasit Charoenkwan, Wen-Lin Huang, Hui-Ling Huang, Shinn-Ying Ho

**Affiliations:** 1 Institute of Bioinformatics and Systems Biology, National Chiao Tung University, Hsinchu, Taiwan; 2 Department of Biological Science and Technology, National Chiao Tung University, Hsinchu, Taiwan; 3 Department and Institute of Industrial Engineering and Management, Minghsin University of Science and Technology, Xinfeng Hsinchu, Taiwan

## Abstract

**Background:**

Protein-protein interactions (PPIs) are involved in various biological processes, and underlying mechanism of the interactions plays a crucial role in therapeutics and protein engineering. Most machine learning approaches have been developed for predicting the binding affinity of protein-protein complexes based on structure and functional information. This work aims to predict the binding affinity of heterodimeric protein complexes from sequences only.

**Results:**

This work proposes a support vector machine (SVM) based binding affinity classifier, called SVM-BAC, to classify heterodimeric protein complexes based on the prediction of their binding affinity. SVM-BAC identified 14 of 580 sequence descriptors (physicochemical, energetic and conformational properties of the 20 amino acids) to classify 216 heterodimeric protein complexes into low and high binding affinity. SVM-BAC yielded the training accuracy, sensitivity, specificity, AUC and test accuracy of 85.80%, 0.89, 0.83, 0.86 and 83.33%, respectively, better than existing machine learning algorithms. The 14 features and support vector regression were further used to estimate the binding affinities (P*kd*) of 200 heterodimeric protein complexes. Prediction performance of a Jackknife test was the correlation coefficient of 0.34 and mean absolute error of 1.4. We further analyze three informative physicochemical properties according to their contribution to prediction performance. Results reveal that the following properties are effective in predicting the binding affinity of heterodimeric protein complexes: apparent partition energy based on buried molar fractions, relations between chemical structure and biological activity in principal component analysis IV, and normalized frequency of beta turn.

**Conclusions:**

The proposed sequence-based prediction method SVM-BAC uses an optimal feature selection method to identify 14 informative features to classify and predict binding affinity of heterodimeric protein complexes. The characterization analysis revealed that the average numbers of beta turns and hydrogen bonds at protein-protein interfaces in high binding affinity complexes are more than those in low binding affinity complexes.

## Background

Protein-protein interactions (PPIs) regulate a wide range of biological processes, involved in almost every cellular function. Majority of the proteins in living cells interact with partner proteins and form a complex to regulate proper functions. PPI employs transport mechanisms, muscle contractions, regulations of gene expression and signal transductions [1,2]. PPIs are classified into different types based on their functional and structural characteristics. According to their stability, interaction surface and involvement, PPIs are classified into obligate and non-obligate, homo and hetero, or permanent and transient [3].

Binding affinity defines the strength of PPIs, and is represented by a dissociation constant (*K_d_*). Binding affinity is crucial in drug developments and therapeutics, and thus, many approaches have been developed to measure the binding affinity. Generally, these approaches are categorized into two groups. The first group identifies the binding affinity using scoring functions and two hybrid systems, surface plasmon resonance and forster resonance energy transfer [4]. These experimental methods for estimating the binding affinity are costly and time consuming. The second group uses computational methods to predict protein binding affinity, such as binding site prediction studies [5-7], empirical scoring function, knowledge based and quantitative structural methods [8-10]. Machine learning models have been developed with structure- and sequence-based features to predict and classify the binding affinities. Yugandhar *et al*. using sequence descriptors to develop a prediction method SMO using support vector machines (SVM) to discriminate high and low binding affinity of heterodimeric protein complexes [11]. Additionally, the works [12,13] used support vector regression (SVR) models with structure-based features to predict binding affinities for different sets of protein complexes. Alternatively, the work [14] used functional features with a SVR to represent the strength of interactions and observed physicochemical and conformational changes. For existing studies of predicting binding affinities, the prediction models work using small datasets. Only few sequence based studies on predicting the binding affinities. This work aims to predict the binding affinities of heterodimeric complexes and characterize the used sequence-based features.

Nearly 4,000 PPIs exist and the growth of PPIs in size increases speedily. It is a challenging task to accurately predict the binding affinities of PPIs based on sequence information only. This work proposes a SVM-based binding affinity classifier, called SVM-BAC, to classify heterodimeric protein complexes by predicting their binding affinity. SVM-BAC using SVM with an optimal feature selection method, an inheritable bi-objective combinatorial genetic algorithm (IBCGA) [15], can identify a small set of features to determine the binding affinity of protein complexes from 580 sequence descriptors including 531 physicochemical properties from the AAindex database [16] and 49 selected physicochemical, energetic and conformational properties of the 20 amino acids from the literature [17]. A dataset with 216 heterodimeric protein-protein complexes is established from the work [11,18]. SVM-BAC identified 14 sequence descriptors to classify the high and low binding affinity of protein complexes and obtained 10-fold cross validation and independent test accuracies of 85.80% and 83.33%, respectively. Using these 14 features selected by SVM-BAC with SVR, we estimated the binding affinity in terms of dissociation constant (P*kd*) for 200 heterodimeric protein complexes and obtained correlation coefficient of 0.34 and a mean absolute error of 1.4. Contribution analysis of prediction has been used to select top-ranked features. The top-two physicochemical properties apparent partition energy [19] and principal component analysis IV [20], and an important secondary structure based feature, i.e. normalized frequency of beta turn [21] are effective in predicting the binding affinity of heterodimeric protein complexes.

## Results and Discussion

### Prediction performance of SVM-BAC

We have classified heterodimeric protein complexes by predicting their binding affinities. A dataset consisting of 108 and 108 complexes with high and low binding affinity was used, respectively. All the sequences were encoded into 580 sequence descriptors. SVM-BAC incorporating with the optimal feature selection algorithm IBCGA selected a set of 14 informative sequence descriptors to discriminate the high and low binding affinity complexes.

SVM-BAC achieved the training (10-fold cross validation), test accuracies and Matthews correlation coefficient (MCC) of 85.80%, 83.33% and 0.71 respectively, slightly better than the SMO method [11] with 76.1%, 83.3% and 0.66, shown in Table 1. SVM-BAC predicted high and low binding affinity complexes with training sensitivity and specificity of 0.89 and 0.83, and test sensitivity and specificity of 0.89 and 0.78, respectively. To avoid the biased results due to the fix partition of training and test datasets, we also evaluated the performance of SVM-BAC using the whole dataset of 216 complexes in terms of 10-fold and 5-fold cross validations (10-CV and 5-CV). The sensitivity, specificity, and accuracy of 10-CV were 0.759, 0.842, and 80.09%, respectively. The sensitivity, specificity, and accuracy of 5-CV were 0.777, 0.842, and 81.01%, respectively. The accuracies of 10-CV and 5-CV using 216 complexes were slightly smaller than the test accuracy (83.33%) on 54 complexes.

**Table 1:** Performance results of SVM-BAC using 162 training and 54 test complexes.

Method	Training 10-CV	SEN	SPE	AUC	Test (n complexes)	SEN	SPE	AUC
SVM-BAC	85.80%	0.888	0.827	0.86	83.33% (54)	0.888	0.777	0.82

SMO [11]	76.1%	0.756	0.767	0.76	83.3% (30)	0.813	0.857	0.84

SEN (Sensitivity), SPE (Specificity) and AUC (Area under the ROC curve)

**Figure 1:**
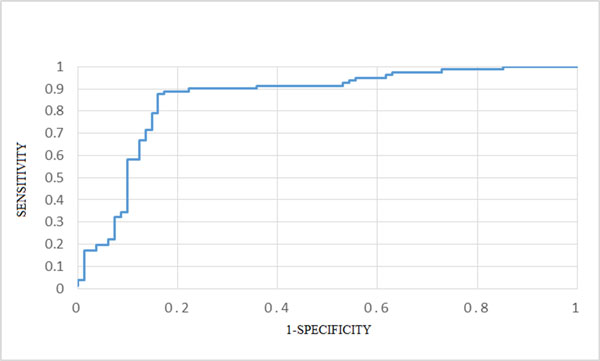
**ROC curve for the SVM-BAC performance evaluation**. The area under the ROC curve (AUC) is 0.86 using the training dataset.

Classifier performance of using the ROC curve is shown in Figure 1. The areas under the ROC curve (AUC) were 0.86 and 0.76 for SVM-BAC and the SMO method, respectively. The SMO method is better than several machine learning algorithms such as Bayesian logistic regression, Naïve Bayes, Multilayer perception, K-nearest neighbors, J48 decision tree, and random forest [11]. The 14 sequence descriptors identified by SVM-BAC are given in Table 2. The results suggest that the 14 features selected by the optimization method IBCGA were effective in predicting the binding affinity of complexes.

**Table 2:** The 14 physicochemical properties identified by SVM-BAC.

Rank	Aaindex_ID	Description	MED
1	GUYH850105	Apparent partition energies calculated from Chothia index [19]	35.18
2	SNEP660104	Principal component IV [20]	32.71
3	RACS820113	Value of theta (i) (Rackovsky-Scheraga, 1982) [40]	31.48
4	MITS020101	Amphiphilicity index (Mitaku et al., 2002) [41]	31.48
5	MAXF760103	Normalized frequency of zeta R (Maxfield-Scheraga, 1976) [42]	27.77
6	CIDH920104	Normalized hydrophobicity scales for alpha/beta-proteins (Cid et al., 1992) [43]	19.13
7	AURR980119	Normalized positional residue frequency at helix termini C"' (Aurora-Rose, 1998) [44]	17.90
8	TANS770103	Normalized frequency of extended structure (Tanaka-Scheraga, 1977) [45]	16.66
9	CHOP780101	Normalized frequency of beta turn (Chou-Fasman,1978a) [21]	12.96
10	PALJ810107	Normalized frequency of alpha-helix in all-alpha class (Palau et al., 1981) [28]	12.96
11	QIAN880116	Weights for beta-sheet at the window position of -4 (Qian-Sejnowski, 1988) [46]	12.96
12	PALJ810110	Normalized frequency of beta-sheet in all-beta class (Palau et al., 1981) [28]	10.49
13	TAKK010101	Side-chain contribution to protein stability (kJ/mol) (Takano-Yutani, 2001) [47]	9.25
14	Nm-Protein	Average medium-range contacts folding [17]	4.32

**Figure 2:**
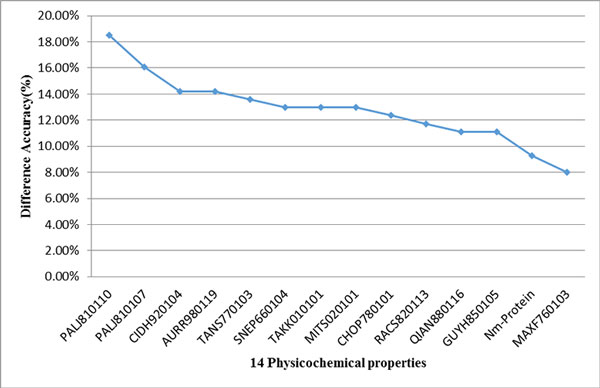
**Difference accuracies of individual physicochemical properties using knock-out analysis**.

Furthermore, we evaluated individual effect of these 14 features on prediction accuracy using knock-out analysis. Removing of an informative feature makes a significant decrease between 8 and 18% in terms of prediction accuracy, shown in Figure 2. These results suggest that the 14 features selected by IBCGA have substantial effects on discriminating high and low binding affinity of protein complexes.

### Estimating binding affinities

**Figure 3:**
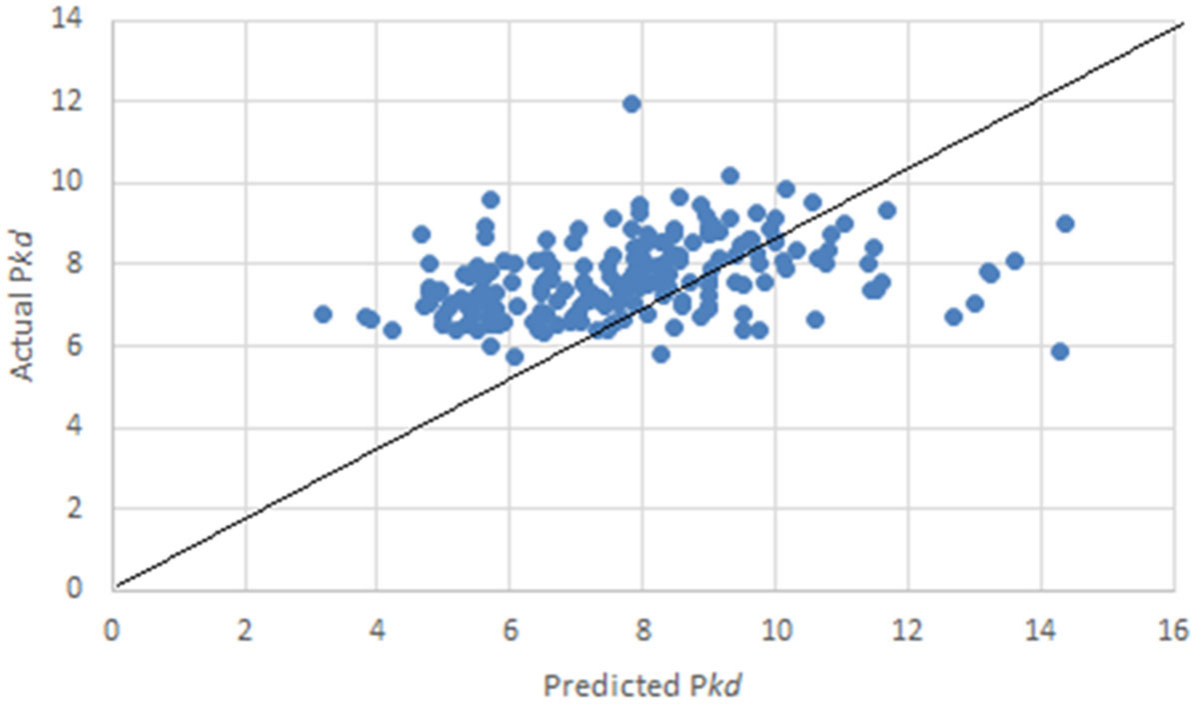
**Estimation performance of jackknife test using the SVR-based method for 200 heterodimeric complexes**.

**Figure 4:**
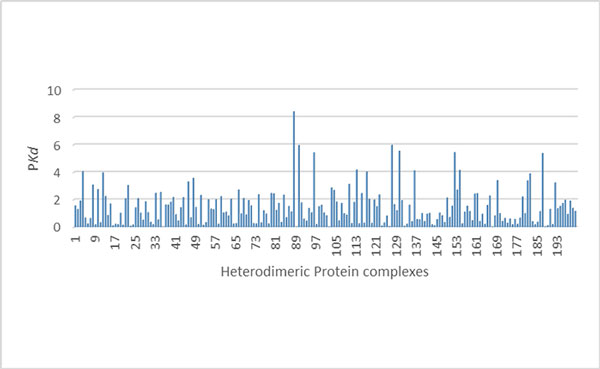
**Mean absolute error of *Pkd *(binding affinity dissociation constant) for 200 heterodimeric complexes**.

Binding affinity of a heterodimeric protein-protein complex is estimated in terms of dissociation constant (P*Kd)*. The binding affinity dissociation constant depends on many factors, such as structural features, interface properties and physiological factors, which are not easily obtained from primary sequences only. We made an attempt to estimate the binding affinities using the promising features of amino acids that were used to predict high and low binding affinity complexes. There were 200 heterodimeric protein complexes used to estimate the binding affinities, which covered various ranges of binding affinity values (P*kd) *and functions. Support vector regression (SVR) was used as a prediction model to estimate binding affinities. Our model was trained using the 14 sequence descriptors and the P*Kd *value. The proposed sequence based model using SVR yielded the correlation coefficient of 0.34 and mean absolute error of 1.4 (Table 3). The correlation result between estimated binding affinities and actual binding ones is shown in Figure 3. Mean absolute error for 200 heterodimeric complexes is shown in Figure 4. The 200 protein-protein complexes and their respective P*kd *values were reported in Additional file 1: Table S1.

**Table 3:** Estimation performance of SVR using Jackknife test on 200 heterodimeric complexes

Estimation method	Features (sequence descriptors)	Coefficient correlation (*R*)	Mean absolute error (*Pkd*)
SVR	14	0.34	1.4

Although we have used an effective set of sequence features, the estimation result of binding affinity was not good enough for the whole dataset, irrespective of their function types. The result was consistent with the recently published prediction method of binding affinity using amino acid sequence feature [22] that they predicted the binding affinity using 642 sequence-based features and obtained poor performance in terms of correlation coefficient on 135 protein complexes. It is noted that protein-protein binding affinities also rely on their function types.

Interestingly, when we observed the estimation error of 200 complexes, we found nearly 150 out of 200 complexes that the mean absolute error was 0.87. The result revealed that amino acid properties are also influential factors to estimate the binding affinities for heterodimeric complexes with specific functional types. However, considering all the 200 complexes with various functional types, the estimation performance was not satisfactory, consistent with the work [14] for the prediction of binding affinity on diverse protein-protein interactions.

Although the promising properties of amino acids can predict high and low binding affinity complexes with satisfactory results, yet they cannot be used to accurately estimate the actual binding affinity dissociation constant. To advance the estimation ability, structural features, interface properties, physiological factors, and partner residues information are useful which are not available from the primary sequences themselves. Partner-aware prediction of interacting residues in protein-protein complexes from sequence information has significance in characterizing the interaction [23].

### Physicochemical property analysis

The top-two physicochemical properties according to the main effect difference (MED) are apparent partition energies calculated from Chothia index (GUYH850105) [19] and principal component IV (SNEP660104) [20]. Large value of MED means the great contribution to prediction performance. An influential secondary structure related property, normalized frequency of beta turn (CHOP780101) [21] was at rank 9. The three physicochemical properties are further analyzed and discussed below. Table 4 presents the values of 20 amino acids for the three physicochemical properties, the amino acid compositions in high and low binding affinity complexes, and amino acid compositional difference between the two classes.

**Table 4:** Amino acid composition (AAC) of high binding affinity (HBA) and low binding affinity (LBA) complexes and three physicochemical properties.

Amino acid	HBA_AAC (%)	LBA_AAC (%)	Composition difference (%)	^a^GUYH850105	^b^SNEP660104	^c^CHOP780101
Ala	7	6.8	0.2	-0.27	-0.062	0.66
Arg	4.2	4.8	-0.6	2	-0.167	0.95
Asn	4.8	4.5	0.3	0.61	0.166	1.56
Asp	5.2	5.9	-0.7	0.5	-0.079	1.46
Cys	2.6	1.7	0.9	-0.23	0.38	1.19
Glu	3.9	4.2	-0.3	1	-0.025	0.98
Gln	5.7	7	-1.3	0.33	-0.184	0.74
Gly	7.8	6.6	1.2	-0.22	-0.017	1.56
His	2.1	2.3	-0.2	0.37	0.056	0.95
Ile	4.7	5.3	-0.6	-0.8	-0.309	0.47
Leu	8.2	9	-0.8	-0.44	-0.264	0.59
Lys	5.7	6.4	-0.7	1.17	-0.371	1.01
Met	1.7	2.3	-0.6	-0.31	0.077	0.6
Phe	3.5	4	-0.5	-0.55	0.074	0.6
Pro	4.9	4.7	0.2	0.36	-0.036	1.52
Ser	8.6	6.7	1.9	0.17	0.47	1.43
Thr	6.7	6	0.7	0.18	0.348	0.96
Trp	1.7	1.4	0.3	0.05	0.05	0.96
Tyr	3.9	3.5	0.4	0.48	0.22	1.14
Val	7	6.9	0.1	-0.65	-0.212	0.5

^a^GUYH850105 = Apparent partition energies.

^b^SNEP660104 = Principal component analysis IV.

^c^CHOP780101 = Normalized frequencies of beta turn.

#### The property of apparent partition energies

The property of GUYH850105 is described as "Apparent partition energies calculated from Chothia index [19]". Chothia index is based on calculating the ratio of buried molar fractions for each amino acid in globular proteins. Guy proposed Salvation energies calculated from vapour pressure of side chain analogues R (ΔSE) which are highly correlated (R = 0.86) with Chothia apparent transfer energy scale [19]. This property describes the buried hydrophobicity in proteins.

**Figure 5:**
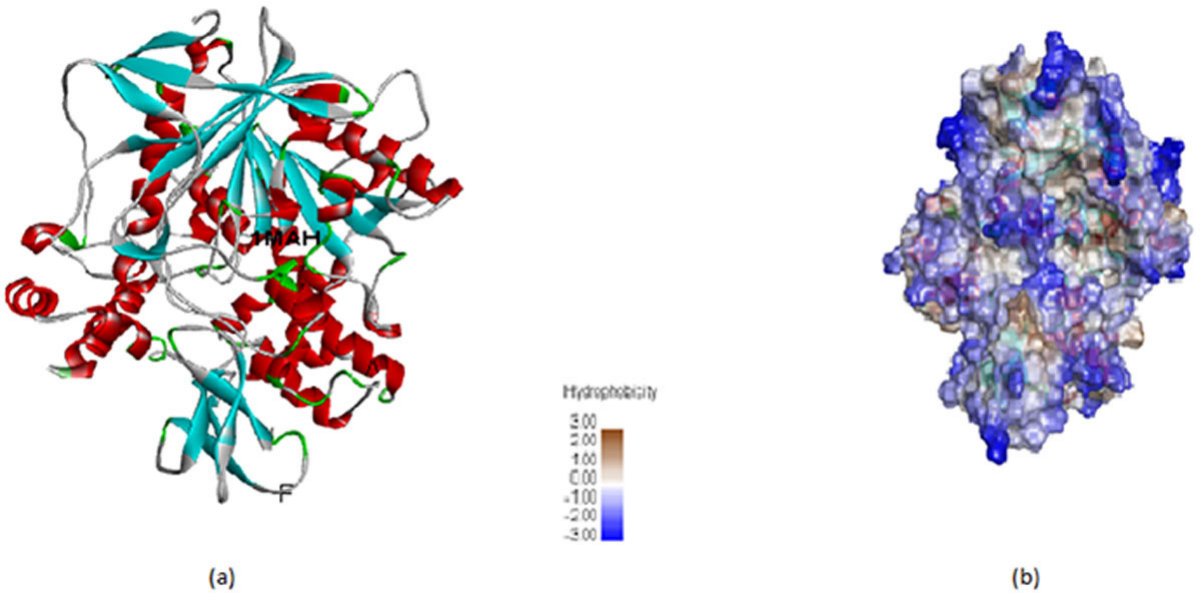
**Surface hydrophobicity of 1MAH**. The color of the surfaces represents the level of hydrophobicity. The blue, white, and brown colors represent low, mediate, and high hydrophobicity, respectively. (a) Secondary structures of 1MAH (b) Surface hydrophobicity of 1MAH. Protein structures are drawn using Discovery studio 4.0.

The buried hydrophobicity nature of protein-protein interactions has been extensively studied. Protein-protein binding directly correlates with total buried hydrophobic surface area and the binding energy increases with the increment of interfacial buried surface area [18]. Mutational studies on free energy change on mutants Δ (ΔG^0^) correlated with hydrophobic buried area. Upon adding hydrophobic buried surface at their interface leads to gaining of free energy Δ (ΔG^0^) = -15 ± 1.2 cal/molA^2^. Statistical and experimental estimations state that the increase of hydrophobic buried surface enhances the protein binding affinity [24,25]. We thus calculated apparent partition energies for hydrophobic amino acids in our dataset according to the property [19]. We found that the average apparent partition energies for high binding affinity complexes are slightly larger than those for low binding affinity complexes that mean of apparent partition energies obtained for high and low binding affinities were -55.10 and -60.87, respectively. This property analysis declared the importance of hydrophobic amino acid residues at buried region which is one of the major influential factors to increase the binding strength of an interaction. Hydrophobic core in high binding affinity complex PDB_ID: 1MAH as an example is shown in Figure 5. The analysis results are consistent with the previous studies of binding affinity.

#### The property of principal component analysis IV

The property of SNEP660104 was described as "Relations between chemical structure and biological activity in principal component analysis IV". Sneath calculated the correlations of amino acids for the use in principal component analysis. Four vectors (Vectors I, II, III and IV) were derived from the 20 amino acid correlations [20]. These four vectors were interpreted as different properties, in which Vector IV represents hydroxythiolation. Hydroxythiolation property has an ability to form hydrogen bonds.

Hydrogen bonds and salt bridges are one of the major contributors to protein-protein interactions. Polar and non-polar side chains significantly contribute to stabilization of the complexes. Polar side chains stabilize the protein complexes through hydrogen bonds. In general, protein interfaces are more hydrophilic than interior residues and form more hydrogen bonds at interfaces [26]. In trypsin-pancreatic trypsin inhibitor, insulin dimer and hemoglobin alpha beta dimer complexes, most of the hydrogen bonds are charged; opposite charges are more favorable to hydrogen bond formation, and 86% of buried polar atoms are favorable to form hydrogen bonds. Chothia et al. reported mean of hydrogen bonds per 100 A^2 ^ΔASA, and maximum and minimum numbers of hydrogen bonds in heterodimeric complexes were 1.89 and 0.29, respectively. Xu-et al analyzed hydrogen bond and salt bridge specificity, and charge distribution at protein-protein interfaces [27].

**Figure 6:**
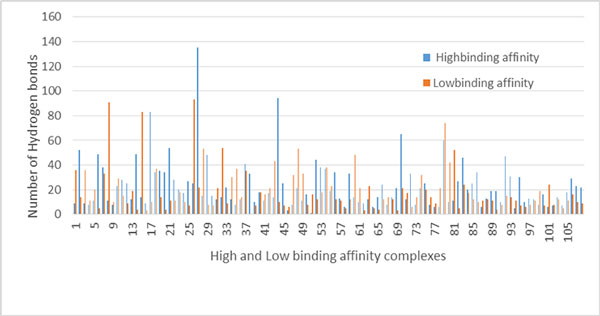
**The numbers of hydrogen bonds at interfaces in heterodimeric complexes**. X-axis denotes the identification numbers of high and low binding affinity complexes. Y-axis denotes the number of hydrogen bonds at interfaces in a protein complex.

We measured the numbers of hydrogen bonds at protein-protein interfaces in high and low binding affinity complexes. The average numbers of hydrogen bonds in high and low binding affinity complexes were 22.83 ± 19.70 and 19.42 ± 17.91, respectively. The hydrogen bonds at their interfaces were more enriched in high binding affinity complexes than in low binding affinity complexes. Contribution of these hydrogen bonds in overall protein-protein interactions is various and crucial to pinpoint. The numbers of hydrogen bonds at interfaces in high and low binding affinity complexes are shown in Figure 6.

#### The property of normalized frequencies of beta turns

The property of CHOP780101 is described as "Normalized frequencies of beta turn". Beta bends/ turn is formed by the polypeptide chain folds back on itself by 180 degrees. Beta turns shows three conformations based on their phi, psi values, and two major types exists i.e., types 1 and 2 [28]. Amino acid preferences are different in each type. In type 2 beta turns Gly possess a major preferences at position i+2 and i+3. Usually, beta turns promote antiparallel beta sheets, which can stabilize the secondary structure and these beta sheets are involved in protein interactions. Beta sheet interactions are involved in the binding of Ras oncoproteins to their receptors, significant part occurred in cell signaling pathway [29,30], immune system, and HIV-1 proteases and inhibitors [31]. Non-regular structures such as turns, helix and loops at interfaces are large groups in heterodimeric complexes and also have large percentages of interface residues at protein-protein complexes belonging to non-regular regions only [32].

**Figure 7:**
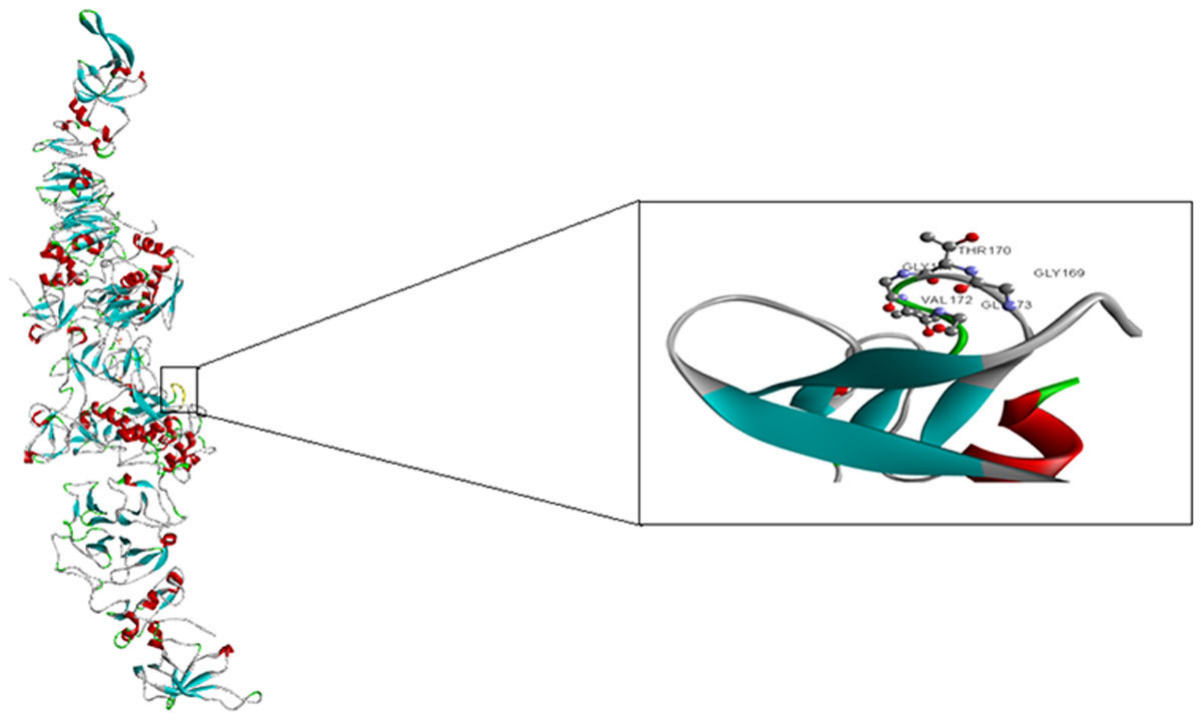
**Structure of PROMMP-2/TIMP-2 COMPLEX (PDB code 1GXD)**. Left: View of the enzyme-inhibitor complex complete structure. Right: A close-up view of type-2 beta turn from the whole complex structure and arrangement of amino acids shown as balls-and-stick model.

To examine the beta turn participation in heterodimeric complexes, we calculated the number of beta turns in the used dataset. Totally, 4,528 beta turns participate in the 216 heterodimeric complexes. On average, every high and low binding affinity complex possesses 23.78 ± 16.89 and 18.27 ± 12.42 beta turns, respectively. Notably, the mean number of beta turns in high binding affinity complexes is significantly larger than that in low binding affinity complexes where the p-value of student's t-test is 0.003. The difference accuracy of the beta turns property CHOP780101 was 12.35% using the knock-out analysis (Figure 2). Though, there are several factors influencing the protein binding affinities, beta turn is one of the most important factors in binding affinity prediction. A better insight into beta turn would have the potential to improve our current protein structure analysis and prediction methods. Beta turn formation in the example complex PROMMP-2/TIMP-2 is shown in Figure 7.

All 14 physicochemical properties and their amino acid composition preferences were calculated for high and low binding affinity complexes, reported in Additional file 2: Table S2.

### Significance of H-bonds and beta turn properties in predicting high and low binding affinity complexes

We estimated the individual effects of H-bonds and beta turn properties in the binding affinity classification by knocking out one of the two corresponding properties, SNEP660104 and CHOP780101. The difference accuracies for the H-bonds and beta turn properties are 12.96% and 12.35%, respectively (Figure 2). Elimination of these two features decreases the overall prediction accuracy of 14.81%. The result suggests that the H-bond and beta turn properties equally contribute to predict high and low binding affinity complexes.

## Conclusions

Characterizing the physicochemical properties influencing the protein binding affinity has a significant role in protein-protein interaction studies. We developed amino acid based predictor named as SVM-BAC to classify the high and low binding affinity complexes by identifying 14 informative properties. Moreover, the SVR-based prediction method using the 14 features was investigated to examine the ability of predicting the binding affinities for the whole set of complexes of various functional types. Our model estimated the binding affinities (P*kd*) of 200 heterodimeric complexes with mean absolute error of 1.4 and it can be further refined by considering the categorization of functional types. Further physicochemical analysis revealed that buried hydrophobicity, beta turns, and hydrogen bonds are influential factors in protein binding affinity. The property analysis would be helpful to understand the underlying mechanism in the protein binding affinities. Though, protein binding affinities depend on various factors, we attempted to find out the contribution of sequence properties in binding affinity prediction.

## Methods

### Datasets

We compiled a dataset of 262 high and low binding affinity complexes from previous literature [11,18]. Protein complexes possess diverse functions and molecular weights. Protein complexes with *Kd *< 10^-8 ^M are regarded as high binding affinity class and those with *Kd *≥ 10^-8 ^are considered as low binding affinity class. We extracted the protein sequences from the PDB database [33]. After removing uncertain entries and deleting the sequences if sequence length is less than 50 amino acids, a balanced dataset contains 216 heterodimeric protein complexes including 108 positive (high binding affinity) and 108 negative (low binding affinity) samples. Since each amino acid is significant and possesses the ability to change binding free energies [34], we did not apply the redundancy criterion to decrease the sequence identity and thus considered all the 216 complexes. We randomly selected 162 samples (75%) as training and 54 samples (25%) as test sets. We utilized 531 amino acid sequence based features from AAindex and 49 properties from literature [16,17].

For estimating the binding affinity value (P*kd*), we used the same 216 heterodimeric complexes which were used to predict the high and low binding affinity complexes. There were 16 complexes removed from the 216 complexes because there is no absolute value of binding affinity available. Finally, 200 protein complexes have been considered for the estimation experiment. Binding affinity values were collected from different sources [35,36]. These binding affinity values are in a diverse range from μM to nM, i.e. from 10^-3 ^to 10^-15 ^M. The 200 complexes possess diverse functional groups such as antibody/antigen, enzyme-inhibitor, enzyme-substrate, G-protein containing, receptor-containing and other enzymes.

### Physicochemical properties

Kawashima and Kanehisa developed the AAindex database which collects numerical indices representing physicochemical and biochemical properties of amino acids [16]. In our work we used 531 physicochemical properties from AAindex and 49 additional features from protein folding related sequence descriptors [17] as candidate features to construct a SVM-based classifier for the discrimination of high and low affinity binding proteins. The original sequences of the selected datasets were transformed into the numerical indices according to the each feature's corresponding values of amino acids. The numerical values of the features were normalized to the scale [-1, 1] for using SVM. When translating into machine learning lexicon for predicting protein functions, variable-length sequences may arise the problem of encoding the feature vector. It is important for classification that the feature vector is formulated into a feature vector with constant length by feature generation. In this work, we used 580 sequence descriptors for encapsulating the global information about proteins of variable length in a fixed length formation. Each of the 580 features was derived from the averaged value of a specific property of amino acids, which was independent of the sequence order of two proteins. The aims of this work are to identify the informative properties of amino acids and then predict the binding affinity in heterodimeric complexes. So, order-dependent sequence features were not used in the proposed method.

The procedure of feature representation for the 580 physicochemical properties is described as follows:

Step 1: Collect the high and low binding affinity sequences from the training dataset.

Step 2: Calculate the composition *w*(*a_i_*) of a complex for the i^th ^amino acid a_i _of 20 amino acids to encode the protein sequence of variable length into the feature vector of length 580.

Step 3: Calculate the feature value of the *p^th ^*physicochemical property, TPCP(*p*), of a protein complex, where *p *= 1, 2, ..., 580.

(1) $$TPCP\left(p\right)=\overset{20}{\underset{i=1}{\sum }}w\left(a_{i}\right).PCP_{p}\left(a_{i}\right)$$

where PCP*_p_*(*a_i_*) is the value of the *a_i _*amino acid of the *p^th ^*physicochemical property.

### Inheritable bi-objective combinatorial genetic algorithm (IBCGA)

In this work, the inheritable bi-objective combinatorial optimization genetic algorithm (IBCGA) [15] is used for the feature selection. The feature selection is a combinatorial optimization problem *C(n, m)*. IBCGA selects a small set of *m *features from a large number of *n *candidate features while optimizing the prediction performance. IBCGA is an efficient global optimization algorithm comprising an intelligent evolutionary algorithm which uses orthogonal array crossover to efficiently solve large parameter optimization problems. The inheritable mechanism can conserve the features that can improve the predication accuracy in the searching procedure.

In using IBCGA, the parameter setting of SVM and feature selection were encoded into binary genes to be optimized simultaneously. In this work, the commonly used genetic algorithm (GA) terms are gene and chromosome, represent as GA-gene and GA-chromosome for the discrimination. The GA-chromosome consists of *n *= 580 binary genes *b_i _*for selecting informative features and two 4-bit GA-genes for tuning the parameters C and γ of SVM. The *i*^th ^property is excluded from the SVM classifier if *b_i _*= 0, otherwise it will be included. This method can encode the 16 values of γ and C ∈ {2^-7^, 2^-6^, ..., 2^8^}. In the SVM classifier, digitalized and normalized protein sequences in the training data set were used as input. In this work, the range of the size of candidate feature set selected by IBCGA is from *r_start _*= 10 and *r_end_*= 20. The feature selection algorithm IBCGA is described as follows.

(Initialization) randomly generate an initial population of individuals.

Step 1: (Evaluation) Evaluate the fitness value of all individuals using the fitness function that is the prediction accuracy in terms of 10-fold cross validation.

Step 2: (Selection) Use a conventional method of tournament selection that selects the winner from two randomly selected individuals to generate a mating pool.

Step 3: (Crossover) Select two parents from the mating pool to perform orthogonal array crossover operation.

Step 4: (Mutation) Apply a conventional mutation operator to the randomly selected individuals in the new population. Mutation is not applied to the best individuals to prevent the best fitness value from deterioration.

Step 5: (Termination test) If the stopping condition (reaching a prespecified number of generations) for obtaining the solution is satisfied, then output the best individual as the solution. Otherwise, go to Step 2.

Step 5: (Inheritance) If *r *<*r_end, _*randomly change one bit in the binary GA-genes for each individual from 0 to 1; increase the number *r *by one, and go to Step 2. Otherwise, stop the algorithm.

### Binding affinity prediction method SVM-BAC

After the feature selection (*m *features) and parameter settings (γ and C) of SVM are done by using IBCGA, the binding affinity prediction method SVM-BAC can be implemented. SVM is an effective method used in the two-class classification and regression problems [37]. SVM works implicitly in the feature space by only computing the corresponding kernel K(*x_i_, x_j_*) between any two objects *x_i _*and *x_j_*:

(2) $$K\left(x_{i},x_{j}\right)=\Phi {\left(x_{i}\right)}^{T}\Phi \left(x_{j}\right)$$

where $\Phi \left(x\right)$ is used as a mapping function. Support vector regression (SVR) has an ability to interpret the property values from a number of samples in high dimensional space. Due to its effective regression abilities, SVR has been used for many biological prediction problems. This work used the following equations to measure the performance evaluation.

(3) $$Accuracy=\frac{TP+TN}{TP+TN+FP+FN}$$

(4) $$Sensitivity=\frac{TP}{TP+FN}$$

(5) $$Specificity=\frac{TN}{TN+FP}$$

(6) $$MCC=\frac{TP\times TN-FP\times FN}{\sqrt{\left(TP+FP\right)\left(TP+FN\right)\left(TN+FP\right)\left(TN+FN\right)}}$$

Where *TP *is true positive; *TN *is true negative; *FP *is false positive; *FN *is false negative; MCC is Matthews Correlation Coefficient.

### Calculation of H-bonds and beta turns

Hydrogen bonds and beta turns were calculated using the PDB sum database [38] and DSSP webserver [39].

## Competing interests

The authors declare that they have no competing interests

## Authors' contributions

Yerukala Sathipati Srinivasulu (YSS) and Shinn-Ying Ho (SYH) designed the system, participated in manuscript preparation, and carried out the detail study. Jyun-Rong Wang (JRW), Ming-Ju Tsai (MJT), Kai-Ti Hsu (KTH), Phasit Charoenkwan (PCW), Wen-Lin Huang (WLH), and Hui-Ling Huang (HLH) participated in the design of the system, implemented programs, and discussed the results. All authors have read and approved the final manuscript.

## Supplementary Material

Additional file 1 Protein-protein complex PDB ids and corresponding P*kd *values. The additional file contains 200 protein-protein complex PDB ids and corresponding P*kd *values. (*.pdf). Click here for file

Additional file 2 **Statistics of the 14 physicochemical properties for 216 heterodimeric complexes**. The additional file contains statistics description for 216 heterodimeric complexes. (*.xls). Click here for file

## Declaration Statement

Publication charge for this work was funded by National Science Council of Taiwan under the contract number MOST-104-2627-M-009-009-, MOST-104-2221-E-009-183-, and "Center for Bioinformatics Research of Aiming for the Top University Program" of the National Chiao Tung University and Ministry of Education, Taiwan, R.O.C. for the project 104W962.

This article has been published as part of *BMC Bioinformatics *Volume 16 Supplement 18, 2015: Joint 26th Genome Informatics Workshop and 14th International Conference on Bioinformatics: Bioinformatics. The full contents of the supplement are available online at http://www.biomedcentral.com/bmcbioinformatics/supplements/16/S18.

## References

[B1] Nooren IMA Thornton JM Diversity of protein-protein interactions Embo Journal 2003 22 14 3486 3492 12853464 10.1093/emboj/cdg359 PMC165629

[B2] Pawson T Nash P Protein-protein interactions define specificity in signal transduction Genes & Development 2000 14 9 1027 1047 10809663

[B3] Keskin O Gursoy A Ma B Nussinov R Principles of protein-protein interactions: What are the preferred ways for proteins to interact? Chemical Reviews 2008 108 4 1225 1244 18355092 10.1021/cr040409x

[B4] Phizicky EM Fields S PROTEIN-PROTEIN INTERACTIONS - METHODS FOR DETECTION AND ANALYSIS Microbiological Reviews 1995 59 1 94 123 7708014 10.1128/mr.59.1.94-123.1995 PMC239356

[B5] La D Kong MS Hoffman W Choi YI Kihara D Predicting permanent and transient protein-protein interfaces Proteins-Structure Function and Bioinformatics 2013 81 5 805 818 10.1002/prot.24235 PMC4084939 23239312

[B6] La D Kihara D A novel method for protein-protein interaction site prediction using phylogenetic substitution models Proteins-Structure Function and Bioinformatics 2012 80 1 126 141 10.1002/prot.23169 PMC3240730 21989996

[B7] Su Y Zhou A Xia XF Li W Sun ZR Quantitative prediction of protein-protein binding affinity with a potential of mean force considering volume correction Protein Science 2009 18 12 2550 2558 19798743 10.1002/pro.257 PMC2821273

[B8] Zhang C Liu S Zhu QQ Zhou YQ A knowledge-based energy function for protein-ligand, protein-protein, and protein-DNA complexes Journal of Medicinal Chemistry 2005 48 7 2325 2335 15801826 10.1021/jm049314d

[B9] Ma XH Wang CX Li CH Chen WZ A fast empirical approach to binding free energy calculations based on protein interface information Protein Engineering 2002 15 8 677 681 12364582 10.1093/protein/15.8.677

[B10] Vreven T Hwang H Pierce BG Weng ZP Prediction of protein-protein binding free energies Protein Science 2012 21 3 396 404 22238219 10.1002/pro.2027 PMC3375440

[B11] Yugandhar K Gromiha MM Feature selection and classification of protein protein complexes based on their binding affinities using machine learning approaches Proteins-Structure Function and Bioinformatics 2014 82 9 2088 2096 10.1002/prot.24564 24648146

[B12] Kastritis PL Bonvin A Are Scoring Functions in Protein-Protein Docking Ready To Predict Interactomes? Clues from a Novel Binding Affinity Benchmark Journal of Proteome Research 2010 9 5 2216 2225 20329755 10.1021/pr9009854

[B13] Ma D Guo YZ Luo JS Pu XM Li ML Prediction of protein-protein binding affinity using diverse protein-protein interface features Chemometrics and Intelligent Laboratory Systems 2014 138 7 13

[B14] Luo JS Guo YZ Zhong Y Ma D Li WL Li ML A functional feature analysis on diverse protein-protein interactions: application for the prediction of binding affinity Journal of Computer-Aided Molecular Design 2014 28 6 619 629 24789327 10.1007/s10822-014-9746-y

[B15] Ho SY Chen JH Huang MH Inheritable genetic algorithm for biobjective 0/1 combinatorial optimization problems and its applications Ieee Transactions on Systems Man and Cybernetics Part B-Cybernetics 2004 34 1 609 620 10.1109/tsmcb.2003.817090 15369097

[B16] Kawashima S Kanehisa M AAindex: Amino acid index database Nucleic Acids Research 2000 28 1 374 374 10592278 10.1093/nar/28.1.374 PMC102411

[B17] Gromiha MM A statistical model for predicting protein folding rates from amino acid sequence with structural class information Journal of Chemical Information and Modeling 2005 45 2 494 501 15807515 10.1021/ci049757q

[B18] Chen JM Sawyer N Regan L Proteinprotein interactions: General trends in the relationship between binding affinity and interfacial buried surface area Protein Science 2013 22 4 510 515 23389845 10.1002/pro.2230 PMC3610057

[B19] Guy HR AMINO-ACID SIDE-CHAIN PARTITION ENERGIES AND DISTRIBUTION OF RESIDUES IN SOLUBLE-PROTEINS Biophysical Journal 1985 47 1 61 70 3978191 10.1016/S0006-3495(85)83877-7 PMC1435068

[B20] Sneath PHA Relations between chemical structure and biological activity in peptides Journal of Theoretical Biology 1966 12 39 10.1016/0022-5193(66)90112-3 4291386

[B21] Chou PY Fasman GD EMPIRICAL PREDICTIONS OF PROTEIN CONFORMATION Annual Review of Biochemistry 1978 47 251 276 10.1146/annurev.bi.47.070178.001343 354496

[B22] Yugandhar K Gromiha MM Protein-protein binding affinity prediction from amino acid sequence Bioinformatics 2014 30 24 3583 3589 25172924 10.1093/bioinformatics/btu580

[B23] Ahmad S Mizuguchi K Partner-Aware Prediction of Interacting Residues in Protein-Protein Complexes from Sequence Data PLoS ONE 2011 6 12 e29104 22194998 10.1371/journal.pone.0029104 PMC3237601

[B24] Vallone B Miele AE Vecchini P Chiancone E Brunori M Free energy of burying hydrophobic residues in the interface between protein subunits Proceedings of the National Academy of Sciences of the United States of America 1998 95 11 6103 6107 9600924 10.1073/pnas.95.11.6103 PMC27592

[B25] Sammond DW Eletr ZM Purbeck C Kimple RJ Siderovski DP Kuhlman B Structure-based protocol for identifying mutations that enhance protein-protein binding affinities Journal of Molecular Biology 2007 371 5 1392 1404 17603074 10.1016/j.jmb.2007.05.096 PMC2682327

[B26] Cherfils J Duquerroy S Janin J PROTEIN-PROTEIN RECOGNITION ANALYZED BY DOCKING SIMULATION Proteins-Structure Function and Genetics 1991 11 4 271 280 10.1002/prot.340110406 1758882

[B27] Xu D Tsai CJ Nussinov R Hydrogen bonds and salt bridges across protein-protein interfaces Protein Engineering 1997 10 9 999 1012 9464564 10.1093/protein/10.9.999

[B28] Palau J Argos P Puigdomenech P PROTEIN SECONDARY STRUCTURE - STUDIES ON THE LIMITS OF PREDICTION ACCURACY International Journal of Peptide and Protein Research 1982 19 4 394 401 7118409

[B29] Avruch J Zhang XF Kyriakis JM RAF MEETS RAS - COMPLETING THE FRAMEWORK OF A SIGNAL-TRANSDUCTION PATHWAY Trends in Biochemical Sciences 1994 19 7 279 283 8048167 10.1016/0968-0004(94)90005-1

[B30] Marshall M INTERACTIONS BETWEEN RAS AND RAF - KEY REGULATORY PROTEINS IN CELLULAR-TRANSFORMATION Molecular Reproduction and Development 1995 42 4 493 499 8607981 10.1002/mrd.1080420418

[B31] Wlodawer A Miller M Jaskolski M Sathyanarayana BK Baldwin E Weber IT Selk LM Clawson L Schneider J Kent SBH CONSERVED FOLDING IN RETROVIRAL PROTEASES - CRYSTAL-STRUCTURE OF A SYNTHETIC HIV-1 PROTEASE Science 1989 245 4918 616 621 2548279 10.1126/science.2548279

[B32] Guharoy M Chakrabarti P Secondary structure based analysis and classification of biological interfaces: identification of binding motifs in protein-protein interactions Bioinformatics 2007 23 15 1909 1918 17510165 10.1093/bioinformatics/btm274

[B33] Berman HM Battistuz T Bhat TN Bluhm WF Bourne PE Burkhardt K Iype L Jain S Fagan P Marvin J The Protein Data Bank Acta Crystallographica Section D-Biological Crystallography 2002 58 899 907 10.1107/s0907444902003451 12037327

[B34] Thorn KS Bogan AA ASEdb: a database of alanine mutations and their effects on the free energy of binding in protein interactions Bioinformatics 2001 17 3 284 285 11294795 10.1093/bioinformatics/17.3.284

[B35] Cheng T Li X Li Y Liu Z Wang R Comparative Assessment of Scoring Functions on a Diverse Test Set Journal of Chemical Information and Modeling 2009 49 4 1079 1093 19358517 10.1021/ci9000053

[B36] Kastritis PL Moal IH Hwang H Weng ZP Bates PA Bonvin A Janin J A structure-based benchmark for protein-protein binding affinity Protein Science 2011 20 3 482 491 21213247 10.1002/pro.580 PMC3064828

[B37] Vapnik VN An overview of statistical learning theory Ieee Transactions on Neural Networks 1999 10 5 988 999 18252602 10.1109/72.788640

[B38] Laskowski RA Hutchinson EG Michie AD Wallace AC Jones ML Thornton JM PDBsum: a Web-based database of summaries and analyses of all PDB structures Trends in Biochemical Sciences 1997 22 12 488 490 9433130 10.1016/s0968-0004(97)01140-7

[B39] Touw WG Baakman C Black J te Beek TAH Krieger E Joosten RP Vriend G A series of PDB-related databanks for everyday needs Nucleic Acids Research 2015 43 D1 D364 D368 25352545 10.1093/nar/gku1028 PMC4383885

[B40] Rackovsky S Scheraga HA DIFFERENTIAL GEOMETRY AND POLYMER CONFORMATION. 4. CONFORMATIONAL AND NUCLEATION PROPERTIES OF INDIVIDUAL AMINO-ACIDS Macromolecules 1982 15 5 1340 1346

[B41] Mitaku S Hirokawa T Tsuji T Amphiphilicity index of polar amino acids as an aid in the characterization of amino acid preference at membrane-water interfaces Bioinformatics 2002 18 4 608 616 12016058 10.1093/bioinformatics/18.4.608

[B42] Maxfield FR Scheraga HA Status of empirical methods for the prediction of protein backbone topography Biochemistry 1976 15 23 5138 5153 990270 10.1021/bi00668a030

[B43] Cid H Bunster M Canales M Gazitua F HYDROPHOBICITY AND STRUCTURAL CLASSES IN PROTEINS Protein Engineering 1992 5 5 373 375 1518784 10.1093/protein/5.5.373

[B44] Aurora R Rose GD Helix capping Protein Science 1998 7 1 21 38 9514257 10.1002/pro.5560070103 PMC2143812

[B45] Tanaka S Scheraga HA STATISTICAL MECHANICAL TREATMENT OF PROTEIN CONFORMATION. 5. MULTISTATE MODEL FOR SPECIFIC-SEQUENCE COPOLYMERS OF AMINO-ACIDS Macromolecules 1977 10 1 9 20 557155 10.1021/ma60055a002

[B46] Qian N Sejnowski TJ PREDICTING THE SECONDARY STRUCTURE OF GLOBULAR-PROTEINS USING NEURAL NETWORK MODELS Journal of Molecular Biology 1988 202 4 865 884 3172241 10.1016/0022-2836(88)90564-5

[B47] Takano K Yutani K A new scale for side-chain contribution to protein stability based on the empirical stability analysis of mutant proteins Protein Engineering 2001 14 8 525 528 11579219 10.1093/protein/14.8.525

[B48] Yeh C-M Liu Y-C Chang C-J Lai S-L Hsiao C-D Lee S-J Ptenb mediates gastrulation cell movements via Cdc42/AKT1 in zebrafish PloS one 2011 6 4 e18702 21494560 10.1371/journal.pone.0018702 PMC3073981

