# Supplementary material for: Characterizing informative sequence descriptors and predicting binding affinities of heterodimeric protein complexes
Source: BMC Bioinformatics. 2015 Dec 9;16(Suppl 18):S14. doi: 10.1186/1471-2105-16-S18-S14 (PMC4682391; doi:10.1186/1471-2105-16-S18-S14)
Supplement: Additional file 1 — Protein-protein complex PDB ids and corresponding Pkd values. The additional file contains 200 protein-protein complex PDB ids and corresponding Pkd values. (*.pdf). [file 1471-2105-16-S18-S14-S1.pdf]

Table S2. Protein-protein complex PDB ids and corresponding P*kd* values

PDB_ID	*Pkd*
1E4K	5.769551
1Y64	8.05061
2FJU	5.275724
1I2m	11.60206
1US7	5.823909
3BZD	7.017729
2HQS	7.568636
2O3B	11.49485
1YCS	7.522879
1AKJ	3.886057
1DQJ	8.552842
1MAH	10.60206
1AVZ	4.79588
1SBB	5.522879
2OMZ	6.39794
2QNA	7.080922
3KUC	6.354578
3DOE	7.69897
1IB1	7.69897
1XDT	8
3K1R	9
2BTF	5.638272
2VIR	9
1KKL	7.346787
1AY7	9.69897
1IQD	10.85387
3GC3	5.677781
1IJK	7.638272
1QA9	5.045757
1GPW	8.30103
1BUH	7.113509
1AVX	9.318759
2VLQ	10.64207
1VET	7.89279
1AK4	4.79588
1AXI	7.853872
1KTZ	6.537602
2OZA	8.60206
1MLC	7.040959
2OOB	4.221849

PDB_ID	*Pkd*
3BN3	4.69897
3OHM	6.69897
2J12	7.69897
1PXV	9.508638
1U0S	6.638272
1EWY	5.443697
1BVN	11.03621
1XG2	8.30103
1NB5	10.16115
3BLH	6.522879
1GP2	6.595166
3CPH	6.481486
1IBR	9
1YVB	8.187087
1LFD	5.721246
1RRP	9.522879
1R0R	10.5376
1E96	5.568636
1RLB	6.09691
1WQJ	6.062984
1NVU	5.443697
1AHW	8.468521
1OPH	8.30103
2AQ3	4.920819
1E6E	6.065502
1JTG	9.39794
3CQC	8.39794
1FLE	9
1MQ8	5.522879
1GLA	4.958607
1XQS	5.187087
1T6B	9.769551
3CX8	6.468521
2SNI	11.69897
1ZM4	5.886057
1Y6K	9
1KXP	9.045757
7CEI	14.30103
1KXQ	9.045757
1BRS	12.69897

PDB_ID	*Pkd*
1A0O	5.69897
1DE4	7.167491
1R8S	3.823909
1WQ1	4.769551
2J0T	9.39794
2VSM	7.455932
1I4D	5.522879
1OP9	9.154902
2NYZ	9.30103
1K5D	9.522879
2SIC	10.14874
1F6M	5.568636
3JZA	11.52288
1NW9	7.886057
3MJ7	5.30103
1WEJ	9.148742
1EER	11.4318
1FAK	6.552842
2HLE	7.39794
1BKD	8.468521
1USU	5.481486
1GHQ	5.366532
2A9K	7.221849
2UUY	8.251812
1JWH	7.886057
2WY8	6.443697
1UUG	13
2MTA	5.346787
1KLU	5.337242
1EMV	13.61979
1EAW	10.30103
3MCA	6.408935
1B6C	6.552842
2P45	6.935542
1ZHI	6.69897
2V8S	4.657577
1BVK	7.853872
2B4J	7.958607
1EFU	7.522879
2HRK	8.045757

PDB_ID	*Pkd*
1CBW	7.958607
1DFJ	13.22915
3SGB	10.74473
2WPT	7.823909
2B42	8.958607
3GQI	7.481486
3KNB	6.026872
2PCC	5.79588
2UYZ	7.086186
1CGI	10.79588
1GXD	8.283997
3NVN	8.026872
1HCF	9.585027
1H9D	7.346787
1HE8	5.49485
2VDB	9.823909
2F4M	7.187087
1F34	10
1JIW	11.39794
1FSK	9.619789
2ABZ	8.552842
3BP8	8.387216
1JMO	6.920819
1ZLI	8.886057
3BS5	7.033858
1A2K	6.823909
2I25	9
1M10	8.236572
3OKY	5.886057
1GRN	6.619789
1RV6	9.769551
1LP1	5.69897
1ACB	9.69897
1XD3	6.522879
1BJ1	8.468521
2JEL	8.552842
1N8O	14.35655
1FQJ	7.173925
3K8P	8
1TMQ	7.958607

PDB_ID	P*kd*
3BEG	7.30103
2WWX	7.102373
1NSN	10
2GOX	8.853872
2AJF	7.79588
2TGP	5.619789
1NCA	8.080922
1S1Q	3.19382
2WO3	5.638272
2FD6	9

PDB_ID	P*kd*
1OC0	9
2V9T	8.086186
1EFN	7.420216
1BGX	9
3FPU	9.920819
2PTC	13.22185
2C0L	6.958607
1WDW	8.60206
1E6J	7.537602
1KAC	7.823909

PDB_ID	P*kd*
2OT3	5.744727
2I9B	9.481486
1HE1	7.638272
1VFB	8.431798
1FC2	7.638272
1H1V	7.60206
1PVH	7.09691
2OOR	7.79588
1J7D	5.69897
1FFW	5.853872

PDB_ID	P*kd*
2OUL	8.769551
1GCQ	4.769551
1JPS	10
1AZS	5
2PCB	5
1EZU	10.09691
1R6Q	6.481486
1Z0K	5.113509
1P2C	10
1ATN	8.69897
